# How older adults express loneliness in everyday life: a qualitative content analysis

**DOI:** 10.1186/s12889-026-30207-0

**Published:** 2026-11-14

**Authors:** Sébastien Lindhagen, Magdalena Kania-Lundholm, Josefin Wångdahl, Jerry Norlin, Kevin J. McKee, Lena Dahlberg

**Affiliations:** 1 School of Health and Welfare, Dalarna University, Falun, Sweden; 2 School of Culture and Society, Dalarna University, Borlänge, Sweden; 3 Aging Research Center, Karolinska Institutet and Stockholm University, Stockholm, Sweden

**Keywords:** Loneliness, Older adults, Indirect expressions, Sweden, Semi-structured interviews

## Abstract

**Background:**

Loneliness among older adults is a significant public health concern, associated with mental and physical health outcomes. Although loneliness is widely studied, less is known about how older adults’ express loneliness in everyday language. Understanding these expressions is important for improving screening instruments and communication strategies. The aim of this study is to describe how older adults express their everyday experiences of loneliness.

**Methods:**

A descriptive qualitative design was employed using semi-structured face-to-face interviews with 16 community-dwelling older adults aged 80 years and above living in Sweden. Participants were purposively sampled from a national panel study based on prior reports of loneliness. Interviews were transcribed and analyzed using inductive manifest content analysis. The analysis focused on the words and phrases that the participants used when they talked about loneliness.

**Results:**

An overarching theme of difficulty in expressing loneliness was identified. Three main categories described how loneliness was expressed: (1) Feelings of loneliness, including descriptions of unpleasant emotions, varying intensity, and fluctuation over time (2). Indirect expressions of loneliness, where participants described loneliness through experiences such as boredom, slowness, emptiness, or through reactions to loneliness such as shame, and resignation (3). Relational expressions, where loneliness was framed in terms of missing social contact, lack of meaningful relationships, and perceived separation from others.

**Conclusions:**

Older adults often express loneliness indirectly, using language that reflects their experiences without explicitly labeling them as loneliness. Approaches that only rely on direct questions of loneliness may therefore overlook older adults who express their negative experience without explicitly naming loneliness. Incorporating indirect expressions into screening, communication, and intervention strategies may improve the detection and support of loneliness in older populations.

## Background

While loneliness affects all age groups, older adults have been shown to be particularly vulnerable [1]. This vulnerability arises partly due to factors associated with later life, such as loss of a partner, a limited social network, a low level of social activity, and poor health [2]. Among older adults aged 65 + in high income countries, a pooled prevalence of approximately 8% experience severe loneliness and around 26% experience moderate loneliness [3, 4], with around 12% of older adults aged 77 + in Sweden report feeling lonely often or almost always [4]. Loneliness poses a significant risk to well-being, increasing the likelihood of mental health issues such as depression and anxiety, as well as physical health problems such as cardiovascular diseases, dementia, and cancer, making it a public health and policy concern [5, 6]. Based on qualitative interviews with Swedish community-resident older adults, this study explores how loneliness is expressed in everyday life.

### Understanding loneliness

Loneliness is a multifaceted phenomenon arising from the complex interplay of social, psychological, and contextual factors [7]. It is most commonly conceptualized as a negative emotional experience that results from a perceived discrepancy between the social relationships one desires and those one actually has, whether in terms of quantity or quality [8]. The World Health Organisation has recently highlighted how loneliness can be located within the broader context of social connection, where an individual’s connectedness can be understood in terms of the structural, functional, and qualitative dimensions of their relationships [9]. Loneliness should be distinguished from social isolation, which refers to the objective absence of social contacts [10], and from solitude, which is a positive perception or experience of being alone that may be voluntarily sought and have beneficial effects [11]. Consequently, individuals may live alone without feeling lonely, while others may maintain numerous social connections yet still experience profound loneliness [12].

While often measured as a unidimensional construct, loneliness has been conceptualised as multidimensional, encompassing social, emotional and existential dimensions [11]. Social loneliness relates to a perceived lack of available and engaging social networks, characterized by a lack of meaningful interactions and social contacts, feelings of boredom and exclusion from a group [13]. Emotional loneliness relates to a perceived absence of close, intimate relationships such as those with a partner or family member [13], and can manifest as feelings of abandonment or lack of recognition from others, which can occur even when one has contact with other people [11]. These two dimensions are conceptually different but may overlap, for example, following widowhood or reduced social activities [7, 12]. Existential loneliness involves feelings of detachment from other people and the world and an awareness of one’s mortality [14, 15], often occurring in connection to a traumatic experience, a life threatening illness, or the end of life [11].

People often find it difficult to describe their feeling of loneliness [16]. Everyday language includes a variety of terms for feelings of distress, making it difficult for individuals to accurately find the words to describe their experience [17]. Furthermore, individuals often perceive loneliness as conceptually diffuse and characterized by both psychological and contextual factors [18]. As noted by Car and Fang [19], certain forms of loneliness, particularly existential, are often difficult to express as older adults describe a sense of separation but without the words to accurately capture it.

The relatively abstract nature of loneliness makes it challenging for research to capture how loneliness is experienced and expressed. Qualitative research has explored how loneliness is experienced and given meaning in later life. Syntheses of qualitative studies have shown that loneliness often is experienced in terms of loss, disconnection, and a lack of belonging [11]. In a systematic review across age groups, loneliness has been described as having emotional, cognitive and perceptual features, and being impacted by personality and identity, as well as related to the absence of specific relationships [18]. Among community-dwelling older adults with reduced mobility, loneliness has been described as a complex and abstract phenomenon that is difficult to articulate [20]. Other qualitative work has shown that older adults may downplay or distance themselves from loneliness by emphasizing busyness or independence, possibly to avoid being perceived as lonely [16]. In a qualitative meta synthesis of research with older adults, loneliness was connected to multiple negative emotions that drained energy and life spirit, as well as a loss of meaningful interpersonal relationships that contributed to negative self-perception [21]. Taken together, this body of research highlights the complexity of loneliness in old age, and how it is contextualized and intertwined with issues of loss, agency and relationships. Much of this research has focused on the latent meaning or the content of the loneliness experience. In contrast, the present study adopts a manifest approach, examining expressions older adults use when talking about loneliness.

Achieving a greater understanding of how loneliness is expressed by older adults is important, both with regard to advancing scientific knowledge of what language is used to talk about loneliness but also to provide a foundation for the development of interventions to reduce loneliness. For an intervention aimed at reducing loneliness to be effective, it must address the form of loneliness the individual is experiencing, and relate to the context of their life [22–24]. While there are several validated instruments for identifying loneliness, not all have been developed within the populations in which they are applied. When considering loneliness in older adults, it is possible such instruments might not be sufficiently sensitive to how loneliness is experienced in that group if they do not employ the appropriate and relevant phrases and terminology older adults use to describe that experience [23–25].

To strengthen the evidence-base for understanding, identifying and reducing loneliness in older adults, the aim of the present study was to describe how older adults in Sweden express their everyday experiences of loneliness. The study was guided by the following research questions: (a) What words and phrases do older adults use when talking about loneliness? (b) How do older adults describe their responses to feeling lonely?

## Method

### Study design

The present study takes the form of secondary data analysis of qualitative data collected through semi-structured, face-to-face interviews with community-resident oldest-old adults, carried out as a sub-study of the Causes and Actions of Late-life Loneliness (CALL) project [26]. The purpose of the CALL sub-study was to explore oldest-old adults’ descriptions of their experiences of, and responses to loneliness.

### Participants

In total 16 community-dwelling older adults aged 80 + years living in Sweden were included in the CALL sub-study. Individuals were eligible if they had reported experiencing loneliness when participating in the 2021 wave of the Swedish Panel Study of Living Conditions of the Oldest Old (SWEOLD) survey [27]. The SWEOLD survey contains the three-item University of California, Los Angeles (UCLA) Loneliness scale, which has a scoring range of 3–9, as well as two single-item questions on how frequently the respondent experiences loneliness, both with labelled, four-point ordinal response scales [28]. Participants where eligible for inclusion if they scored six or above on the UCLA and/or indicated experiencing loneliness at least ‘Often’ on one or both single-item questions. From those eligible, a purposive selection was made to obtain a sample with variation in gender, co-habitation status, place of residence (urban or rural area) and functional ability. Exclusion criteria were cognitive impairment, living in a care home or other institutions, and difficulty communicating in Swedish. We excluded participants with cognitive impairment due to concerns about their ability to understand and provide appropriate responses to cognitively challenging questions on their experience of loneliness; older adults living in care-homes because their experience of loneliness could be influenced by their distinct social context; and non-Swedish speakers because the project did not have the resources to provide interpreters, and the challenge presented for the analysis of how loneliness is expressed when the data is in different languages.

### Materials

The interview guide used in the CALL sub-study was developed by the CALL research team with input from (a) an international panel of three experts in gerontology and loneliness research, one from the United Kingdom, one from Germany, and one from Finland, and (b) a stakeholder group formed of three representatives of different senior citizens’ organizations and one representative of a humanitarian organization, all based in Sweden. At separate meetings for the expert panel and stakeholder group, the draft interview guide was presented by the CALL research team. Advice and suggestions from both workshops were noted and later considered by the research team. A few additional questions and prompts were added, and some original questions re-phrased. With these revisions completed, the guide was prepared for piloting. The guide was pilot tested on two eligible participants, with only minor adjustments subsequently made to question and prompt order, but none to question and prompt wording. The finalized guide consisted of four sections: *introduction and icebreaker*,* general and individual experience of loneliness*,* individual reasons for loneliness*, and *strategies to reduce loneliness.* See Appendix 1 for the full interview guide.

### Procedure

Recruitment to the CALL study followed an iterative process. One member of the CALL research team (LD) carried out an initial selection of potential participants from the SWEOLD dataset, using the eligibility and sampling criteria described in the subsection above. A list containing only the potential participants’ dataset IDs was then sent to the principal investigator of the SWEOLD study, who then extracted their names and contact details from the dataset. These names and contact details were provided to the CALL researchers who would carry out the interviews (JN and MKL) Potential participants were contacted by letter with information on the study and received a subsequent phone call from one of the interviewers, to answer any questions about the study. Those potential participants agreeing to be part of the study then provided written consent. Once all participants from the initial selection had been interviewed, a new selection was made following the same procedure as described above. This process continued until the interviewers indicated that data saturation had been reached after 16 completed interviews. The interviews were carried out between January and July 2024 in locations chosen by the participants, typically in their homes, except for two participants who preferred another location. The interviews lasted on average 57 min (range 28–108 min). All interviews were audio-recorded and later transcribed verbatim and then pseudonymized by the interviewers.

### Data analysis

Prior to commencing data analysis, the suitability of the interview data for addressing the present study’s research questions was evaluated via an initial read-though of the transcripts by four of the authors (SL, JN, KJM and MKL). Once the suitability of the interview data had been established, the transcripts were analyzed using manifest content analysis, following the procedures described by Elo and Kyngäs [29]. The analysis focused on the words and phrases that the participants used when they talked about loneliness. The present study’s research team have a combined expertise in social gerontology, public health and qualitative research on ageing and loneliness. The interdisciplinary background shaped the study’s research questions, analysis and presentation of the results.

Two researchers (SL and MKL) read the transcripts multiple times to become familiarized with the material, each participant, their personal context and expressions. Thereafter the material was organized using an inductive approach. SL wrote notes directly in the text while reading the transcripts. Notes were taken on the broad topic of loneliness every time loneliness was discussed by the participants or raised as a question.

The notes were given content-characteristic names and grouped under subcategories to reduce the number of categories. Notes and subcategories were compared for similarities and grouped into progressively more abstract categories. This process was carried out by SL under the supervision of two other members of the research team (JW and MKL). As *subcategories* where created, the next process involved abstraction of categories by grouping *subcategories* into *generic categories*. Abstraction of categories involved an iterative process in which categories were repeatedly checked against the coded material, the original transcripts and the notes. Some notes were dismissed due to the realization that they did not relate to the loneliness experience. The process of abstraction was led by SL in discussion with JW and MKL. The *generic categories* were grouped into *main categories*. Lastly, all *main categories* were represented in the *overarching category*. Categorizations were reviewed in discussion with all co-authors on multiple occasions. To enhance the credibility of the analysis, coding decisions and disagreements were discussed in the earlier phases with JW and MKL, and with all co-authors in later stage phases.

All quotes presented were translated from the original Swedish language into English by a Swedish speaker with proficiency in English (SL). The translated quotes were reviewed by a native English speaker (KJM) with proficiency in Swedish, to ensure accuracy and preserve meaning and nuance.

The results are presented in alignment with the Consolidated criteria for Reporting Qualitative research (COREQ) [30].

## Results

Participants’ characteristics are presented in Table 1. Participants were 80 to 92 years of age (M = 86.4), the majority was female (*n* = 12), widowed (*n* = 12), resided in an urban area (*n* = 11), and lived alone (*n* = 12).

**Table 1:** Characteristics of participants

Characteristic	*N*	%
Age (years)		
Mean age	86.4	
Range	80–92	
Gender		
Female	12	75
Male	4	25
Residential setting		
Urban area	11	69
Rural area	5	31
Civil status		
Widowed	12	75
Married	2	12.5
Divorced	2	12.5
Living arrangement		
Living alone	13	81
Living with partner	2	13
Living with adult children	1	6

The analysis resulted in three main categories and an overarching category as presented in Table 2. Below, both the overarching category, main categories, and their constituent generic and subcategories are described in detail.

**Table 2:** Categories derived from the content analysis

Overarching category	Main Category	Generic Category	Subcategories
Difficult to talk about	Feelings of loneliness	Unpleasant feelings	
		Changes over time	• Degrees of loneliness
			• Fluctuating intensity
	Indirect expressions of loneliness	Life as slow or boring	• Loneliness as boring
			• Loneliness as slowness
			• Emptiness or silence
		Managing the situation	• Not possible to manage
			• Possible to manage feelings
			• Resignation
		Self-reflection	• Shame or guilt
			• Self-pity
	Relational expressions	Social connections	• Friends and acquaintances are gone
			• Lack of close relationships
			• Missing people
			• Being alone
		Responses from others	• Rejected by others
			• Gratitude toward others

### Difficult to talk about

The overarching pattern across all interviews concerned the difficulty for participants to describe and articulate the experience of loneliness clearly. Instead of talking about loneliness, participants often talked indirectly about the feeling or experience. Many attempts to explain the experience ended up with participants almost giving up. Sometimes, participants would try to explain their loneliness through stories of past experiences. Other times, participants expressed loneliness as “*it is a little hard to define*” or “*I don’t really have the words for it*, while others said, “*loneliness*,* it is just loneliness*”, or that they “*don’t know*” what loneliness feels like.

” … sometimes I feel that loneliness is so big, its… It’s not possible to explain it, I just can’t explain.” – Participant 6.

#### Feelings of loneliness

Within this category, participants talked about loneliness through the emotional state of the feeling itself. They would try to capture what loneliness felt like, highlighting how it feels to experience loneliness. Participants talked about loneliness through references to two generic categories: Unpleasant feelings; and Changes over time.

When participants tried to describe what loneliness felt like, they often used words such as “*difficult*” or “*hard*”. Most participants used a variety of expressions to express their difficult feelings such as “*more than I would want*”, while others described it as “*gets a little desolate*” or “it’s *awful to be alone*”. Some participants also used short phrases such as “*loneliness was not good*” or “*it is a problem*” when talking about what loneliness felt like. Taken together, participants used a wide variety of expressions to talk about the unpleasant feelings of loneliness.

“… that’s how I feel, desolate. When I wake up in the morning, I feel, oh, now it’s going to be a long day.” – Participant 10.

Participants used different modifiers to describe the intensity with which they experienced loneliness. The descriptions of loneliness indicate that the participants experienced different degrees of loneliness. Several participants used modifiers to amplify the word, such as “*it [loneliness] was terrible*,”, “*total loneliness*”, and “*feels really lonely*”. Modifiers where also used to indicate small degrees of loneliness, with expressions such as “*feeling pretty lonely*”, “*I’m a little lonely”*.

“Yes, now and then. Right now, it’s probably the very worst…”– Participant 11.

Loneliness could also be a constant feeling or fluctuate over time. A few participants talked about loneliness as something constant and expressed it as “*in a way*,* a normal state*”. Other participants expressed it as something that could come “*from time to time*” or that “*It comes and goes*”. The fluctuation of loneliness was expressed as something that could happen “*every now and then*”, or “*appear*” at any time.

” … I can’t say that it’s any particular situation. It comes and goes, it can appear at any time during the day”. – Participant 5.

#### Indirect expressions of loneliness

In this category, participants talked about loneliness without explicitly describing the emotional experience. Instead, loneliness was expressed through its qualities, relation to participants’ situation, or statements about how they managed the feeling. These statements describe the everyday manifestation of loneliness, rather than the emotional experience of loneliness. This main category included three generic categories: Life as slow or boring; Managing the situation; and Self-reflection.

Some participants described loneliness as something that was slow, using words such as “*sometimes I think it’s slow*” or “*it was going so slowly*”. For some participants these terms would be used to describe the lonely feeling of being alone at home or having little to do. In those cases, slowness was used to describe loneliness in relation to the tempo of their day, or the feeling of time passing slowly.

“I guess that’s the way it is. It gets slow sometimes when you’re alone all the time, it does.” – Participant 9.

Some participants talked about loneliness as something “*boring*”, or “*dull*”. This is closely related to statements of loneliness as “*slow*”, but instead highlights lack of stimulation or activity, rather than the pace of time. Participants would talk about loneliness and express it as “*it is boring*” or say, “*it’s boring*,* plain and simple*,* loneliness is boring*”. Participants used these phrases when talking about long days or of times when nothing happened. These sorts of statements would signal that loneliness was present but articulated through other descriptions. Others referred to loneliness indirectly in sentences about everyday life such as “*not fun to eat alone*”.

“Then it would become lonely, and then it would become boring.” - Participant 13.

Many participants described loneliness as “*emptiness*”. Participants would also describe it in terms of “*it felt so empty*” while waiting for the phone to ring or “*come home*,* it feels empty*” when talking about coming home and not having anyone there. Sometimes similar words were used to describe loneliness such as “*at night and it’s completely silent*” or in terms of nothingness such as “*nothing has happened and nothing is going to happen*”. The terms emptiness, silence and nothingness could be used interchangeably in the participants’ descriptions of loneliness and sometimes used as metaphors for loneliness:

”And I myself, am involved with the [voluntary organization]. I’m a bit older there. But I feel that I can go there if it gets a bit empty.”– Participant 12.

Participants described dealing with loneliness in different ways. They talked about loneliness as “*it wouldn’t have been easy*” to manage, or that they “*cannot be alone*” as in unable to stand the feeling. Some participants expressed it as “*don’t know how to manage*” the feeling. These phrases were often framed as not being able to manage the feeling of loneliness. One participant described why they found it difficult to manage the feeling of loneliness.

“Yes, I had, he’s no longer alive, an older brother. But he had already moved out of the house by then. And because my father was so struck by this death, the [my] loneliness felt doubled in a way, you almost became afraid, wondering how he would get through it. How could I, how was I going to manage it?” – Participant 3.

On the contrary, some participants talked about handling or mastering loneliness. Some participants talked about loneliness as something that could be dealt with. They could describe it as “*I can sort of manage it*”, or “*I try to force myself*” to do things, which would stop them from feeling lonely. Other participants talked about loneliness as something they avoided, then they would describe it as “*I’m very active*” or not give it “*time to think about feeling lonely*”. Lastly, some participants expressed loneliness as something they had come to terms with, often describing it as “*but I’ve accepted it*” of their situation, or that they “*did not need to have people*” around them.

“And that has meant that in recent months I have felt incredibly lonely. I can master loneliness. If it’s inside, as I said earlier, that’s fine. I do what I want.” – Participant 5.

Several participants talked about loneliness in ways that made it seem as if it were a burden, something that weighed them down and exhausted them. When they spoke about loneliness, they expressed hopelessness or talked about resignation. Some participants used shorter phrases such as “*given my life away*”, or “*not going anywhere*”” to describe the experience. While others used phrases such as “*it feels like life is running out*”. These expressions sometimes framed loneliness as a feeling that was unrelenting.

“… maybe this is the end, maybe I can’t take it anymore. And should I just lie here and wait for death?” – Participant 8.

A few participants talked about their experience of loneliness through expressions of shame or guilt, sometimes using the word explicitly “*whole thing about shame*”, while others expressed it as “*I won’t do that*”. Some participants would use phrases such as “*only got myself to blame*”, or “*I’m not really that lonely*,* but I still get those feelings sometimes*”, as in talking about loneliness and being guilty of their situation. Others also expressed it as feeling sorry for themselves for their situation.

“Sometimes you can sit there and feel sorry for yourself. But then I think, damn, you´re good, why should you feel sorry for yourself?” – Participant 9.

#### Relational expressions

Many participants talked about loneliness in terms of the absence of social contacts or not having people around them. Expressions in this main category concerned who was available, who was missing and how relationships were distant or lacking. The main category included two generic categories: Social connections; and Responses from others.

Participants would use short phrases such as “*no one left*” to describe the absence of people. They also talked about loneliness as “*do not have anyone to talk to*” or “*everyone is gone*”. These expressions were used when participants described that they no longer had people they knew living nearby, or in relation to the social connections they were used to. Most often participants expressed having no one around as something that had previously existed. In other cases, participants talked about a general sense of disconnect from their community such as “*people I used to know have either moved away or died*”. Loneliness was sometimes described as wanting friends or acquaintances to be able to “*drop in when passing*”.

”Loneliness, it is just loneliness. It’s the absence of social companionship.”– Participant 8.

Many participants talked about loneliness as lacking close or meaningful relationships. They used phrases such as wanting “*someone besides*” to describe the feeling of not having anyone by their side. These absent relationships were important because participants wanted to feel like someone “*really care*” about them, or having “*someone you can share everything with*”.

“Even though there are ups and downs, you always have someone there beside you. But I don’t have that now… It’s someone I could always talk to, or ask, or say something to…” – Participant 14.

When participants talked both about a general sense of wanting people around them, or wanting someone that really cared for them, they would commonly use the word “*missing*”. Participants would say “I *miss certain people very much*”, “*this kind of secure friendship that I miss*” or just a general sense “*missing”* people. These expressions would often be used to frame loneliness as a longing for something that they had before.

“Yes, it’s difficult to know what to say about loneliness… it’s a feeling of missing” – Participant 16.

Participant would use the word “*isolated*” to describe what loneliness was like in relation to other people. These expressions framed loneliness as a perceived separation from other people or from social life. Some would describe loneliness as being “*in the big house*,* all on my own*” or “*I feel trapped*” in their home. These sorts of statements framed loneliness as distance or disconnect from others, rather than a feeling itself.

”Yes, that’s how it is. Otherwise, you become isolated. And then it [loneliness] gets worse, when you’re isolated and have no one.” – Participant 9.

Participants related their experience of loneliness to the role of other people. A common description among participants was the feeling of being rejected by others. This was described as “*people do not invite me over anymore*” or that others “*never get in touch*”. Most often, these descriptions were framed as other people not doing enough to invite or contact them. Some participants described it as “*no one comes here*”, as in physically not coming to the participants’ home. Others expressed this rejection through the phone “*no one calls me anymore*” as in keeping contact without the physical presence.

“No, as I said, I feel very lonely. I don’t know how to define it. Well, it’s just that the phone never rings. It used to ring. We spent a lot of time on the phone and stuff. But now, no.” – Participant 7.

Some participants talked about a general feeling of exclusion from others. This was commonly expressed when there was no particular person to blame for their feelings. Participants talked about feeling “*excluded from the community*”, feeling like “*I was an outsider*”, or “*I don’t feel at home*” in different settings. A form of self-imposed exclusion could also arise, in which participants talked about not connecting with younger generations, stating “*you can’t mix with the next generation*” because they “*have their own lives*”.

“I have to say that I want to… [but] I don’t want to be the one who pushes. It’s usually [grandchild] who calls. Because, I think, he has his own life and doesn’t have time for that.” – Participant 15.

While talking about loneliness, some participants framed their experience as a form of gratitude towards others if they took the time to socialize with the participants. These expressions highlight how the experience of loneliness can shift depending on others’ engagement. Some talked about being rescued from loneliness “*you’re* [interviewer] *here now*,* so this day is saved*”, or that other people were “*kind to spend time with me*” or “*they’re sweet and kind*” if they cared about the participants. This could also be expressed as other people being “*thoughtful*,* that he cares.*” or considerate if they socialized with them.

“… no, it works quite well. We call each other. He’s [old colleague] really nice and keeps in touch. So that’s positive. He’s probably the only person outside [family/immediate circle] that I have.” – Participant 15.

## Discussion

The aim of this study was to explore how older adults’ express loneliness in their everyday lives. Results from the study highlight the difficulty in talking about loneliness explicitly. While some descriptions of the feeling were provided, loneliness was more often expressed through indirect expressions such as boredom, emptiness or shame. When the feeling was discussed, it was described as fluctuating, unpleasant and with varying degrees of intensity. Indirect expressions of loneliness came in a wide range of forms. These expressions were used to talk about the everyday manifestation of loneliness in a descriptive or summative fashion, not loneliness as an emotional experience. Participants talked about loneliness as being slow or boring, or stating that loneliness was a burden. Loneliness was also talked about through their response to the experience, expressed as how participants managed, or could not manage their experience, such as by mastering or not being able to stand the feeling. Lastly, participants talked about loneliness in relation to other people, often through accounts of missing social contact, lacking meaningful relationships, or separateness from others. Taken together, the results suggest that loneliness is not only expressed as an emotional state, but also through indirect expressions of the everyday manifestation of what loneliness is like, such as boring, slow and empty.

Our findings resonate with previous qualitative research showing that loneliness is a complex and abstract experience that is often difficult to articulate [16, 20]. While prior studies have conceptualized loneliness as containing elements of sadness, boredom, fear, emptiness, loss and guilt [18], our findings demonstrate that similar terms are used by older adults to describe what loneliness is like. Talking about boredom can thus be understood as a way of communicating loneliness indirectly, using words and phrases that are more socially acceptable. This aligns with earlier work suggesting that everyday language provides overlapping terms for distress, making it difficult to label loneliness directly [17]. Some participants expressed loneliness as something that could “*come and go*”, while others spoke of it as a “*normal state*”. These expressions relate to research that has demonstrated how loneliness can have both transient and chronic forms, a fluctuating experience for some people and a stable state for others. Transient loneliness can be a temporary response to a life event, while chronic loneliness persists despite changes in life circumstances [31, 32]. While previous research has emphasized the difficulty of articulating loneliness, our findings suggest that indirect expressions are not only a consequence of this difficulty, but also a primary way in which loneliness is communicated for older adults. Similarly, previous research has suggested that older adults may distance themselves from loneliness by emphasizing activity or independence [16, 26]. Results from this study highlight how these strategies are expressed as efforts to keep busy or as forcing themselves to be active, and how they become indirect expressions of loneliness without explicitly naming it. Difficulties in talking about loneliness and attempts to distance oneself from the label ‘lonely’ could also be a consequence of the stigma attached to loneliness, which can often be perceived as a sign of weakness or experienced as failure [21, 33, 34]. That loneliness is associated with stigma may also be reflected in the way participants sometimes expressed their experience of loneliness in terms of shame or guilt.

Research has linked loneliness to the loss of meaningful relationships and a diminished sense of belonging [11, 21, 35], and located it in the context of social connections [9]. Our findings align with this understanding of loneliness and demonstrate how these experiences are expressed through situational phrases where participants refer to the absence of other people rather than to abstract notions of disconnection. This was expressed through phrases such as having *“no one around*” or “*no one left*”. These findings suggest that the loss of meaningful relationships is communicated through words and phrases that make the experience recognizable without directly naming loneliness.

A useful way to understand expressions in this study is through frameworks that distinguish social, emotional, and existential loneliness. Although participants did not use these labels themselves, several patterns in the data correspond with them. Describing the absence of everyday contact or companionship, such as “*no one left*”, or “*the phone never rings*”, align with social loneliness and a lack of broader social networks [13]. Longing for someone who “*really cared*” or wanting “*someone beside*” reflects emotional loneliness, which concerns the absence of close, meaningful relationships [13]. Descriptions such as “*it feels like life is running ou*t” or exclusion from others such as by disconnect from community, or “*I don’t feel at home”* connect to existential loneliness. Often associated with detachment, fundamental separateness from others, or feelings of being left behind by life events [11, 14, 15]. Distinguishing between these dimensions and subsequently their different forms of expressions may support more sensitive screening by helping practitioners identify unmet need, particularly when loneliness is expressed indirectly rather than explicitly.

### Implications

The findings have theoretical, practical and methodological implications for understanding loneliness among older adults. Theoretically, the study findings support conceptualizations of loneliness as multi-dimensional [7], and an experience that varies between and within individuals in terms of its intensity and/or transient or chronic nature [31, 36]. Loneliness was also expressed in relation to other people, which aligns with the framework of social connection [9]. Participants described relationships in structural terms through the absence of social contacts and shrinking social networks, in functional terms through the lack of companionship, support or someone who cared for them, and in qualitative terms through experiences of rejection and exclusion. These findings illustrate how older adults communicate loneliness through perceived deficits across all three dimensions of social connection.

For health and social care practitioners engaged in identifying and understanding loneliness in older adults, the study’s findings highlight the importance of paying attention to the expressions they use to talk about their everyday life, as loneliness may be communicated indirectly, without explicit use of the term ‘lonely’. Such indirect expressions as “*no one calls anymore*”, “*no one left*”, or “*it is boring*” may reflect attempts to avoid the stigma associated with loneliness [21], and/or a difficulty in expressing a complex emotional experience. Use of such expressions could indicate that the practitioner should explore further whether loneliness is present through follow-up investigations, with the caveat that such indirect experiences may not reflect loneliness per se but e.g., declining health, problems with mobility, or other circumstances that limit social engagement. Such an approach aligns with other strategies e.g., the Canadian Coalition for Seniors’ Mental Health Conversation Starter, which encourages practitioners to discuss risk factors for loneliness when working with older adults [37].

Methodologically, the findings have implications for the development of instruments to identify and measure loneliness. The study highlights the words and expressions older adults themselves use to describe and communicate loneliness, which may be valuable when designing survey items and interview guides, i.e., informing decisions regarding what questions to ask and how that should be phrased so that they match more precisely onto older adults’ own experiences. Furthermore, the finding that older adults often have difficulty in talking about loneliness directly has implications for whether direct or indirect approaches should be used in questionnaires and surveys, where direct questions may fail to identify loneliness at the individual level, leading to an underestimation of the prevalence of loneliness in the older adult population [38].

### Strengths and limitations

A strength of this study is the use of semi-structured interviews combined with manifest content analysis to examine how loneliness is expressed in everyday life. This approach captures the participants’ own words, making it possible to understand how loneliness is communicated in everyday use, rather than how it is interpreted by researchers. Using the manifest approach aligns with the aim of exploring how older adults’ express loneliness, knowledge that can be relevant in practical settings, where individuals may not explicitly label themselves as lonely. Another strength of the study is the use of semi-structured interviews with older adults who had previously reported loneliness. This approach, in combination with an interview guide that highlighted different areas of loneliness, assisted the participants in talking about their experience from a wide range of viewpoints, providing rich material on emotional, relational and situational experiences. Lastly, to increase the credibility of the analysis, coding and categorizations were done iteratively by frequently checking the categories against the original data. The categorization was discussed extensively with co-authors on multiple occasions, including the researchers who conducted the interviews for CALL. This procedure ensures that interpretations were not based on a single researcher’s perspective. To enhance confirmability several shorter quotations across all categories were embedded in text verbatim, to allow the reader to draw their own conclusions from the analysis. Furthermore, variation in speech patterns was addressed by presenting quotes from a range of participants, and not just the most articulate. All interviews were conducted in Swedish, and the quotes were later translated into English. Although care was taken to preserve the contextual meanings of the quotes, there is a risk that some nuance in language and expression may have been lost in the process.

By focusing on the actual words spoken by participants, a manifest content analysis may overlook deeper latent meanings or underlying emotional nuances that are likely important when considering loneliness. This approach may oversimplify the complexity of emotional expressions and overlook the broader social context that shapes how older adults talk about loneliness. Findings from this study should thus be understood in the context of how older adults express themselves about loneliness, rather than providing knowledge on the psychological or emotional depth of loneliness. The sample consisted of older adults in Sweden who had previously reported loneliness. Although this approach allowed for a deep exploration of loneliness expressions, the study’s findings may not be transferable to other age groups, or to cultures that differ markedly from Swedish culture, where different linguistic and social norms will shape how loneliness is experienced and expressed [39, 40]. There are other issues relating to the transferability of the study’s findings. First, women formed the majority the sample (75%). Although women have a higher prevalence of loneliness when compared to men [41], a more balanced sample in terms of gender would have been preferable, and it is possible that our findings may not transfer to contexts where men form a majority. Second, we excluded older adults with cognitive impairment, those living in care homes, and non-Swedish speakers. Importantly, these groups could be anticipated to be particularly vulnerable to loneliness. Furthermore, the relatively small sample size limited our ability to examine variations in expressions across subgroups of, e.g., civil status or living arrangement. Future research should explore and compare how the members of different groups express their experiences of loneliness.

### Conclusion

Results from this qualitative manifest content analysis study indicate that older adults aged 80 + in Sweden often find it difficult to express what loneliness feels like. Instead, loneliness is often communicated through indirect expressions such as boredom, slowness, emptiness, or shame, and through relational expressions such as missing social connection, lacking meaningful relationships, or separateness from others. The results suggest that assessment and support initiatives that only rely on direct questions about loneliness risk overlooking older adults who express the negative experience without explicitly naming loneliness. Screening instruments, communication strategies, or interventions may include indirect expressions of loneliness to improve the identification or communication about loneliness. Future research should test whether expression-informed screening tools and communication training improve detection and responsiveness to loneliness across settings.

## Data Availability

All data analysed in this study are not publicly available due to sensitive nature of the interview material and risk of identification of participants. Additional data may be available to from corresponding author upon resonable request and with permission from Swedish Ethical Review Authority.
